# Compressive creep test rig for thermoplastic samples

**DOI:** 10.1016/j.ohx.2025.e00687

**Published:** 2025-08-14

**Authors:** Seyyed Saeed Vaezzadeh, Alireza Godsi Chafjiri, Victoria Nguyen, Xochitl Ramirez, Robert Kelley Bradley

**Affiliations:** a Lamar University, Department of Industrial and Systems Engineering, P.O. Box 10032, Beaumont, TX 77710, USA; b Lamar University, Biology Department, P.O. Box 10037, Beaumont, TX 77710, USA; c Lamar University, Department of Civil & Environmental Engineering, P.O. Box 10024, Beaumont, TX 77710, USA

**Keywords:** Creep, Viscoelastic, Materials testing, Thermoplastics, Student project, Education

## Abstract

Creep occurs when a material is subjected to a constant load resulting in deformation. Thermoplastics are prone to creep and understanding creep behavior is important for predicting the long-term performance of thermoplastic parts that must bear load. Creep is a slow process and measuring it can take days, weeks, months, or longer. Accelerated methods to estimate creep behavior exist, but long-term testing produces the most accurate results. We have developed a creep test rig made from off-the-shelf parts that require minimal machining and is easy to operate. The instrument can provide students at the high school, community college, and university level with a simple entry point to materials research. The long run time means that multiple students can collaborate to acquire the data. The instrument helps expand the overall capacity of the scientific community to generate compressive creep data while simultaneously offering a low-cost way to engage students in research. The instrument can be used for traditional university, government, and industry research. Users may wish to operate the instrument in an air-conditioned space with minimal change in temperature and humidity, but some of the authors have recently published work demonstrating that creep measurement under changing ambient temperature and humidity can provide useful data as well (Vaezzadeh et al., 2025).

## Highlights

• Full instructions for building a compressive creep testing instrument.
• Materials testing of thermoplastics to study viscoelastic properties and creep.
• Appropriate for student projects and education as well as for research.
• Avoid contact friction and shear to produce accurate results.
• Segmented creep in low density polyethylene can be observed.

## Graphical abstract

**Figure:**
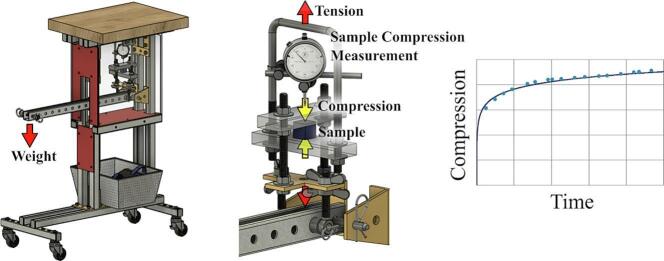

**Specifications table**

See Table 1.

**Table 1:** Specifications.

Hardware name	Creep test rig
Subject area	Engineering and materials science
Hardware type	Measuring physical properties and in-lab sensors
Closest commercial analog	Commercial Lever Arm Uniaxial Compressive Creep Test Machines
Open source license	CERN-OHL-P-2.0
Cost of hardware	$2,200
Source file repository	Mendeley Data https://doi.org/10.17632/6zpxp6rxsx.1See reference 2 for full bibliographic information.

## 1 Hardware in context

The design of the creep test rig is similar to various commercially available and custom-made machines [[3], [4], [5], [6], [7], [8], [9]]. The design is based on ASTM standards D2990 [10] and uses a lever arm to apply a static load to the sample with interlinked plates to convert tension from the lever arm into compression of the sample between the plates. The upper plate moves relative to the lower plate so compression can be monitored by measuring the displacement of the upper plate with a dial indicator or similar measuring instrument. Additional sensors can be added such as a load cell to confirm the force on the sample remains constant and temperature and humidity sensors to monitor atmospheric conditions that the sample is exposed to during the run.

Asyraf et al. reviews a number of custom rigs designed for compressive, tensile, and flexural creep, most utilizing weights and/or lever arms [8]. Some rigs are intended for specific applications such as measuring creep in thin thermoplastic composite tubes [7], fluid-filled polymer foams [6], and investigating alternative creep measurement strategies [5]. Some rigs use direct uniaxial force, either by applied weights [6] or a hydraulic or pneumatic cylinder [4]. A more common approach is the use of a lever arm to apply higher loads via mechanical advantage [3,8]. Lever arm systems have an advantage because they apply loads directly to the sample, with minimal contact points between moving parts. Contact points can produce friction which results in a lower applied load on the sample than expected. Due to the geometry, lever arm rigs can result in slightly varying load as the lever pivots, some rigs, including ours, utilize a leveling mechanism to compensate [9]. Temperature also plays a role in creep measurement, and some rigs include temperature control for the sample [3,4], but precise temperature control can be expensive to implement. All rigs measure displacement or travel of the compression plates or tension clamps, which can be recorded using manual analog or digital equipment. Some rigs also monitor the applied load to ensure it is constant during the run [4,7].

The rig described here is designed to be built from off the shelf components with minimal machining while offering good performance. Features such as load monitoring and temperature control or not included in the base design described here but those interested in incorporating such features can easily adapt the design.

## 2 Hardware description

The rig described in this article was designed and built in a market where parts designated with imperial units are usually less expensive, and a wider variety of parts with imperial units are available. Where units of measure are used to designate a part that was made for imperial units, the imperial units are given with SI units in parentheses. When actual measurements are given, we provide the measurements in SI units with imperial units in parentheses. Original measurements were specified in imperial units and the conversion of fractional units, to SI units sometimes produces a large number of decimal places; these do not necessarily indicate the level of precision required for the measurement but are retained in order to match the fractional values.

The creep test rig is primarily constructed from 1 to 5/8″ × 1–5/8″ (4.1275 cm × 4.1275 cm) strut channel, which is readily available in a variety of lengths and at a relatively low cost. Strut channel provides a strong frame that can be assembled quickly with just a few hand tools. The self-leveling mechanism is a pair of strut channel trolleys that serve as the upper anchoring point for the sample fixture assembly. The trolleys ensure that the force transmitted through the sample fixture assembly is always parallel to the force of gravity acting on the lever arm; the configuration ensures that the sample fixture assembly does not tilt as the lever arm moves, which would result in changing load with respect to the motion of the arm. Creep is a phenomenon that occurs under constant load, so the self-leveling mechanism is an important component of the design. The sample itself serves as the force coupling between the upper and lower halves of the sample fixture assembly, thus ensuring all the load is applied to the sample.

An acrylic enclosure around the sample fixture assembly keeps users and bystanders away from potential pinch-points during a run and prevents accidental contact that could disrupt the experiment. The frame is mounted on caster wheels for easy transport, features a desktop where data logging equipment can be placed, and includes an undercarriage basket for storing extra weights. Various weights can be used to apply load on the lever arm, but our design calls for 10 lb (4.536 kg) steel shot bags that are easy to transport and reduce the chances of injury if dropped on feet. The entire creep test rig measures 96.6216 cm × 45.72 cm x 121.92 cm (3.17ft × 1.5ft × 4ft) and requires a floor space of approximately 106.68 cm × 106.68 cm (3.5ft × 3.5ft).

**Fig. 1:**
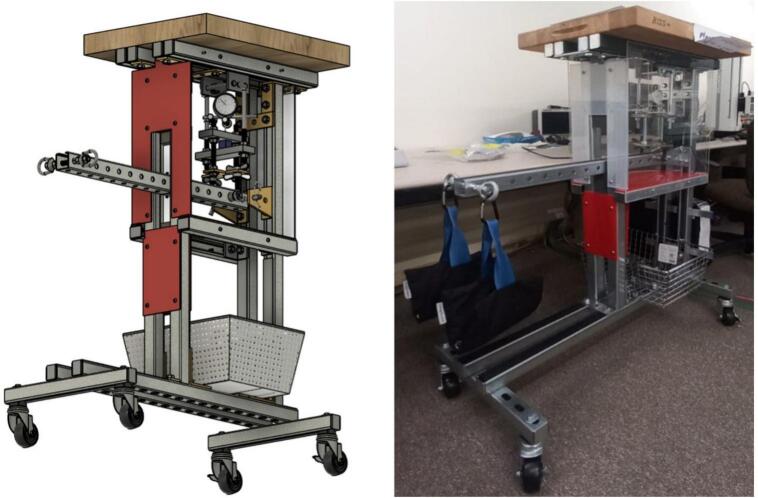
A side-by-side comparison of the CAD model and assembled creep test rig.

Commercial creep test machines have some form of thermal insulation and temperature control. Some small benchtop instruments intended for demonstration or education have enclosures around the sample such that an ice pack or heat pack can be added to change the temperature of the sample. Research-grade instruments are larger and have enclosures with better thermal insulation designed to not just change the temperature but to closely regulate the temperature over the course of the run. The time–temperature superposition principle can be employed to estimate creep behavior at room temperature based on creep at elevated temperature isotherms. Creep occurs faster at elevated temperature, and the principle posits that time and temperature influence creep in a similar way, *i.e.* the compression due to creep over a short time and high temperature can be transformed into the equivalent amount of compression but over a longer time at a lower temperature. This accelerated method of testing has some limitations since it requires certain assumptions to hold true, but the speed of the test runs makes it an attractive alternative to long-term testing. All the commercial, research-grade lever arm creep test machines that the authors are aware of include temperature regulation. The creep test rig described here does not have temperature regulating capabilities; instead, it relies on regulation of the temperature of the room, but it is much less expensive than commercial machines. While benchtop demonstration machines are also less expensive, the creep test rig described here offers a more rugged design capable of handling higher loads and very long runs. The creep test rig can be seen in Fig. 1.

### 2.1 Frame

**Fig. 2:**
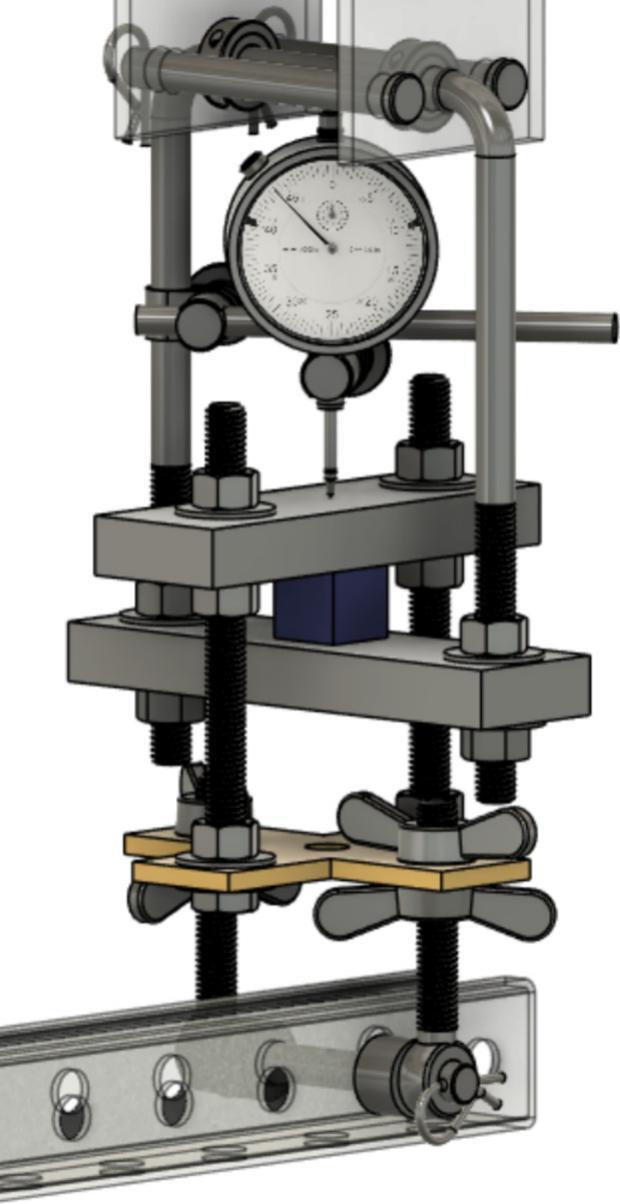
Sample Fixture Assembly.

The frame is primarily constructed from strut-channel and strut-channel hardware. The frame is rugged and relatively heavy, so it incorporates caster wheels for easy relocation. A butcher block desktop provides space for ancillary equipment that may be associated with the creep test rig such as add-on electronics, sensor loggers, signage, etc., but it is not intended for user contact during a run because generated vibrations could interfere with data collection. A wireframe basket is attached to provide storage for the weights when not in use (Fig. 2).

### 2.2 Sample fixture assembly

**Fig. 3:**
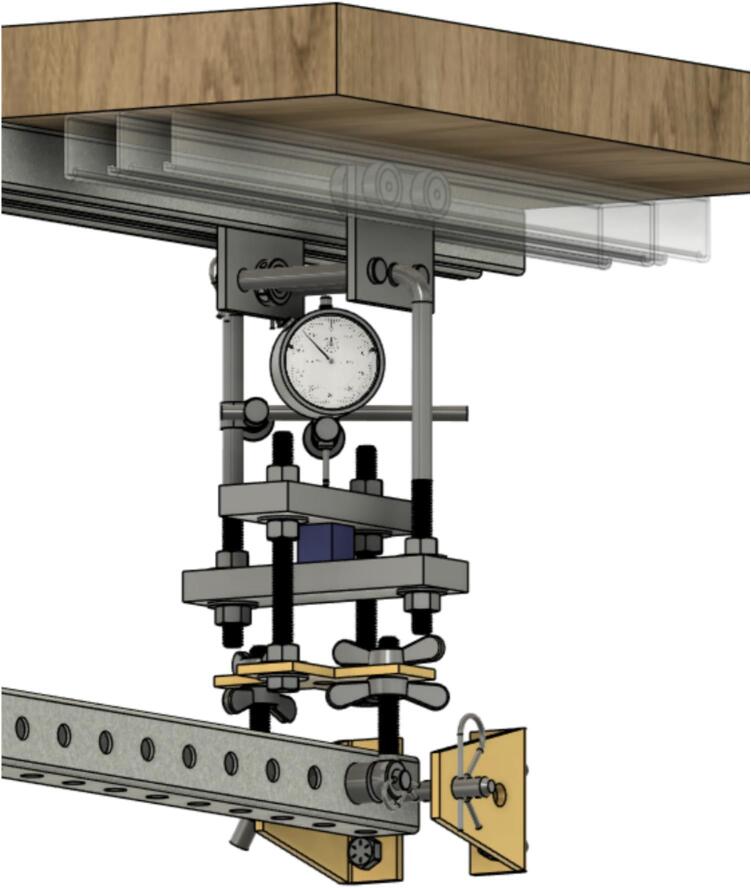
Self-Leveling Mechanism. One of the upper side-by-side strut channels has been rendered semitransparent to show the roller mechanism of one of the two trolleys.

The Sample Fixture Assembly (Fig. 3) consists of two interlinked plates between which the sample is placed. This configuration converts tension applied to the Sample Fixture Assembly into compression applied to the sample. The Sample Fixture Assembly consists of the sample itself, a U-bolt attached to the lower plate, the upper plate attached to a pair of bolts that connect with a flat cross bracket, and a pair of threaded end bolts that connect the assembly to the lever arm via a clevis pin, and the various associated fasteners and hardware. A dial indicator is attached to the U-bolt and measures the movement of the upper plate as the sample is compressed. The entire Sample Fixture Assembly behaves like a beam under tension, connected between the lever arm and the upper portion of the creep test rig frame. The self-leveling mechanism described below ensures that the Sample Fixture Assembly remains upright during a run and does not tilt forward or backward.

### 2.3 Lever arm

The lever arm serves as a force multiplier that allows a relatively small set of weights, in the form of steel shot bags, to apply a very high load to the sample. The creep test rig uses a class-2 lever with a 15-fold mechanical advantage. As long as the Sample Fixture Assembly remains upright or parallel to the direction of gravity, the load applied to the sample will be 15 times the weight of the shot bags added to the lever. The lever arm, which has evenly spaced holes along its length, can accommodate different hanging positions for the shot bags to adjust the mechanical advantage if desired.

### 2.4 Self-leveling mechanism

If the Sample Fixture Assembly were attached to a single pivot point at the top of the frame, it would have to tilt forward and/or backward to accommodate the motion of the lever arm. For the tension on it to remain constant, however, it must stay parallel to the direction of the force of gravity, and therefore, tilting must be prevented. The self-leveling mechanism (Fig. 3) consists of a pair of channel strut trolleys that can roll back and forth within the channel strut frame. The Sample Fixture Assembly is attached to the trolley, and instead of tilting as the lever arm moves, the trolley rolls to maintain the shortest distance between the linkage point on the lever arm and the upper frame that the trolley rolls within. The mechanism automatically keeps the Sample Fixture Assembly upright regardless of the position of the lever arm.

### 2.5 Sample enclosure

Laser-cut acrylic sheets, foam inserts, and strut channel covers are used to enclose the sample within the frame. The enclosure is not intended to be airtight, but it greatly reduces the air pathways in and out of the sample chamber. The enclosure also keeps operators and bystanders away from possible pinch points near the lever arm pivot and prevents direct contact with the Sample Fixture Assembly during the run.

### 2.6 Creep sensor

The creep measurement sensor is attached to the U-bolt within the Sample Fixture Assembly and measures the movement of the upper plate as the sample compresses beneath it. The basic build that is described here uses a dial indicator as the creep measurement sensor, but other options are described later.

### 2.7 Temperature and humidity sensors

Creep measurement is sensitive to changes in temperature. Since this creep test rig is not designed with the capability to self-regulate the sample chamber environment, the rig must depend on the ambient conditions in the room where it is located. Because of the long run times of creep tests, even in a well-regulated room, the possibility of power failure or other anomalies could result in changes of temperature and humidity that should be logged. Temperature and humidity measurement can be achieved through off-the-shelf sensors with built-in data logging.

### 2.8 Use cases

The creep test rig described here can be used:

• To add an inexpensive compressive creep measurement capability to a materials lab.
• To create a relatively low-cost creep testing farm by building multiple rigs and housing them in unused spaces.
• To engage high school, community college, and university students in research. Groups of students can easily learn how to collect data and can create a data set to study and present over the course of a semester or academic year.
• As a base to further develop the capabilities of the rig by adding additional sensors and environmental control capabilities.

## 3 Design files summary

See Table 2.

**Table 2:** Design files.

Design file name	File type	Open-source license	Location of the file
*CAD Model of the Creep Test Rig Assembly*	CAD file	CERN-OHL-P-2.0	Available via Mendeley Data https://doi.org/10.17632/6zpxp6rxsx.1See reference 2 for full bibliographic information.
Laser Cutter Patterns for the Enclosure Acrylic Sheets	SVG files	CERN-OHL-P-2.0	Available via Mendeley Data https://doi.org/10.17632/6zpxp6rxsx.1See reference 2 for full bibliographic information.

The CAD Model of the Creep Test Rig Assembly depicts how the off-the-shelf components are assembled to make the rig. The CAD model focuses on the placement of components, not on dimensions, because most components are used as-is without machining. Measurements needed for machining specific parts are included in the assembly section; in the CAD model, the parts are shown after these steps are completed. The most involved machining step is laser cutting the acrylic sheets that make up the enclosure around the sample. The Laser Cutter Patterns for the Enclosure Acrylic Sheets include a set of.SVG files that can be used directly or easily converted for use with a laser cutter.

## 4 Bill of materials summary

In the Number column, the first number is the units to be purchased and the second number in parentheses is the number of parts to be used.

See Table 3.

**Table 3:** Bill of materials.

Designator	Component	Number	Cost per unit − currency	Total cost − currency	Source of materials	Material type
ID1	45YV98 Strut Channel − Solid Wall: Steel, Pre-Galvanized, 12 ga Gauge, 3 ft Overall Lg, Silver	4 (4)	$28.10	$112.40	Grainger	Metal
ID2	45YV26 Strut Channel − Slotted: Steel, Pre-Galvanized, 12 ga Gauge, 18 in Overall Lg, Silver	2 (2)	$16.40	$32.80	Grainger	Metal
ID3	45YV28 Strut Channel − Slotted: Steel, Pre-Galvanized, 12 ga Gauge, 3 ft Overall Lg, Silver	2 (2)	$28.10	$56.20	Grainger	Metal
ID4	45YV96 Strut Channel − Solid Wall: Steel, Pre-Galvanized, 12 ga Gauge, 18 in Overall Lg, Silver	2 (2)	$16.40	$32.80	Grainger	Metal
ID5	4A985 Spring Nut: 1/2 in-13 Thread Size, 1/2 in Bolt Size, Steel, Galvanized, Pack of 25	3 (54)	$51.67	$155.01	Grainger	Metal
ID6	4A987 Channel Bolt: 1/2 in-13 Thread Size, 1/2 in Bolt Size, Steel, Galvanized, Pack of 25	2 (50)	$26.80	$53.60	Grainger	Metal
ID7	WB242604 John Boos RA03 − Maple Cutting Board 24″ x 18″ x 2–1/4″	1 (1)	$200.95	$200.95	Global Industrial	Organic
ID8	20715A84 Dial Plunger-Style Variance Indicator	1 (1)	$94.68	$94.68	McMaster-Carr	Other
ID9	4025T121 Steel Tote Basket	1 (1)	$26.03	$26.03	McMaster-Carr	Metal
ID10	3252N3 10 lbs. Weight Bag Filled with Stainless Steel Shot	8 (8)	$46.94	$375.52	McMaster-Carr	Other
ID11	8560K194 Clear Scratch- and UV-Resistant Cast Acrylic Sheet, 24″ × 24″ × 1/8″	2 (2)	$24.26	$48.52	McMaster-Carr	Polymer
ID12	8560K257 Clear Scratch- and UV-Resistant Cast Acrylic Sheet, 12″ × 24″ × 1/8″	1 (1)	$20.89	$20.89	McMaster-Carr	Polymer
ID13	8505K742 Red Cast Acrylic Sheet, 12″ × 24″ × 1/8″	3 (3)	$19.25	$57.75	McMaster-Carr	Polymer
ID14	8643K482 Super-Cushioning Polyurethane Foam Sheet, 3/4″ × 2″ × 36″	1 (1)	$12.73	$12.73	McMaster-Carr	Polymer
ID15	3626T11 Strut Channel Trolley, 4 Wheels	2 (2)	$31.91	$63.82	McMaster-Carr	Metal
ID16	33125T421 Strut Channel Corner Bracket, 90-degree, 4 Holes, Trapezoidal	2 (2)	$12.25	$24.50	McMaster-Carr	Metal
ID17	3138T31 Telescoping Strut Channel, Zinc Plated Steel, 5ft	1 (1)	$101.20	$101.20	McMaster-Carr	Metal
ID18	33125T34 Strut Channel Corner Bracket, 90-degree, 4 Holes	8 (8)	$6.19	$49.52	McMaster-Carr	Metal
ID19	33125T72 Strut Channel Straight Bracket, 3 Holes	8 (8)	$4.62	$36.96	McMaster-Carr	Metal
ID20	2356T11 Strut Channel Caster	2 (2)	$19.58	$39.16	McMaster-Carr	Other
ID21	2356T14 Strut Channel Caster, with brake	2 (2)	$19.32	$38.64	McMaster-Carr	Other
ID22	92320A591 18–8 Stainless Steel Unthreaded Spacer 1″ OD, 5/8″ Long, for 1/2″ Screw Size	2 (2)	$6.92	$13.84	McMaster-Carr	Metal
ID23	90369A122 Stainless Steel Adjustable Bent-Pull Clevis Pins, 1/2″ Diameter, 6″ Long	1 (1)	$14.20	$14.20	McMaster-Carr	Metal
ID24	33125T82 Strut Channel Cross Bracket, 5 holes	1 (1)	$8.54	$8.54	McMaster-Carr	Metal
ID25	97245A444 Zinc-Plated 1004–1045 Carbon Steel Clevis Pin 1/2″ Diameter, 4–1/4″ Usable Length	1 (3)	$13.78	$13.78	McMaster-Carr	Metal
ID26	92865A736 Medium-Strength Grade 5 Steel Hex Head Screw Zinc-Plated, 1/2″-13 Thread Size, 6″ Long, Fully Threaded	2 (2)	$3.51	$7.02	McMaster-Carr	Metal
ID27	5148A18 Indicator Swivel Clamp with Knob Adjustment, 1/2″ Diameter × 3/8″ Diameter	1 (1)	$22.81	$22.81	McMaster-Carr	Other
ID28	89535K871 Multipurpose 304/304L Stainless Steel Rod 3/8″ Diameter, 1/2ft	1 (1)	$3.20	$3.20	McMaster-Carr	Metal
ID29	5148A17 Indicator Swivel Clamp Steel with Knob Adjustment, 3/8″ Diameter × 3/8″ Diameter	1 (1)	$21.27	$21.27	McMaster-Carr	Other
ID30	3060T193 Square U-Bolt Zinc-Plated, Steel, 1/2″-13 Thread Size, 5″ Inside Width, 8–5/8″ High	1 (1)	$12.29	$12.29	McMaster-Carr	Metal
ID31	3798K25 Fully Threaded Rod End Bolt Right Hand 1/2″-13 Shank, 3–1/2″ Shank Center Length	2 (2)	$9.93	$19.86	McMaster-Carr	Metal
ID32	3019T18 Galvanized Steel Eye Nut − for Lifting 1/2″-13 Thread Size	2 (2)	$8.92	$17.84	McMaster-Carr	Metal
ID33	95475A738 Low-Strength Threaded Rods, Zinc-Plated Steel, 1/2″-13 Thread Size, 6″ Long, packs of 1	1 (1)	$4.67	$4.67	McMaster-Carr	Metal
ID34	95462A033 Pack of 100 Medium-Strength Steel Hex Nut Grade 5, Zinc-Plated, 1/2″-13 Thread Size	1 (16)	$29.99	$29.99	McMaster-Carr	Metal
ID35	93441A474 Pack of 10 Slotted Unthreaded Spacers Zinc-Plated Low-Carbon Steel, 21/32″ OD, 0.512″ ID, 1/2″ Long	1 (2)	$9.89	$9.89	McMaster-Carr	Metal
ID36	3611T6 Side-to-Side Stacked Strut Channel Solid, Zinc-Plated Steel, 2ft	2 (2)	$42.38	$84.76	McMaster-Carr	Metal
ID37	3312T82 Zinc-Plated Steel Plug for 1–5/8″ High Strut Channel	20 (20)	$1.80	$36.00	McMaster-Carr	Metal
ID38	3204T12 Strut Channel Cover 5 Feet Long, Black Plastic, for 1–5/8″ Wide Channel	2 (2)	$10.55	$21.10	McMaster-Carr	Metal
ID39	33125T32 Strut Channel Bracket 90 Degree, Zinc-Plated Steel, 2–1/4″ Length, 1–5/8″ Width	4 (4)	$3.70	$14.80	McMaster-Carr	Metal
ID40	8975K776 Multipurpose 6061 Aluminum 1/4″ Thick × 1–1/4″ Wide	1 (1)	$8.52	$8.52	McMaster-Carr	Metal
ID41	90876A450 Wing Nut Steel, 1/2″-13 Thread Size	4 (4)	$7.46	$29.84	McMaster-Carr	Metal
ID42	92141A033 Pack of 50 18–8 Stainless Steel Washer for 1/2″ Screw Size, 0.531″ ID, 1.25″ OD	1 (16)	$7.67	$7.67	McMaster-Carr	Metal
ID43	2380K17 Clamping Two-Piece Shaft Collar for 1/2″ Diameter, Zinc-Plated 1215 Carbon Steel	2 (2)	$9.60	$19.20	McMaster-Carr	Metal
ID44	97493A125 Pack of 10 Partially Threaded Steel Stud M6 × 1.00 mm Thread Size, 16 mm Long	1 (4)	$8.46	$8.46	McMaster-Carr	Metal
ID45	98685A840 Brass Phillips Decorative Rounded Head Screws for Wood, Number 10 Size, 1″ Lon, 25 Pack	1 (4)	$11.40	$11.40	McMaster-Carr	Metal
ID46	92949A540 18–8 Stainless Steel Button Head Hex Drive Screw 1/4″-20 Thread Size, 3/4″ Long, pack of 50	1 (34)	$8.95	$8.95	McMaster-Carr	Metal
ID47	92865A716 Medium-Strength Grade 5 Steel Hex Head Screw, Zinc-Plated, 1/2″-13 Thread Size, 1–1/2″ Long, 25 pack	1 (8)	$17.03	$17.03	McMaster-Carr	Metal
ID48	98335A135 Cotter Pin, 1/2″ with 1/8″ wire, 25pk	1 (3)	$7.35	$7.35	McMaster-Carr	Metal
ID49	9116K619 Annealed 4140 Alloy Steel Bar, 3/4″ Thick, 2″ Wide, 13″ long	1 (2)	$67.34	$67.34	McMaster-Carr	Metal

Parts in the CAD model are titled with the designator, followed by the catalog number and a short description of the part. For example, ID49-9116K619 Annealed 4104 Steel.

## 5 Build instructions

Tools and consumables needed to assemble the creep test rig include wrenches, pliers, Phillips screwdrivers, a drill with a 3/16-inch (0.47625 cm) drill bit, manual tapping tools with a 7/32-inch (0.555625 cm) tap, and cutting oil. Some minimal machining is required, including cutting metal pieces, drilling, tapping, and laser cutting acrylic sheets. Metal cutting can be done with a hacksaw, but using a metal shop band saw will make the process much easier. Drilling part ID49 should be done with a drill press; other drilling operations will require a hand drill. Appropriate PPE should be used during machining and assembly. Before starting the assembly, certain components need to be prepared:

### 5.1 Component preparation

The 5-foot (152.4 cm) strut (ID17), the 1–7/8″ × 1–7/8″ (4.7625 cm × 4.7625 cm) telescoping strut channel with circular holes should be cut into two 76.2 cm (2.5-foot) sections.

**Fig. 4:**
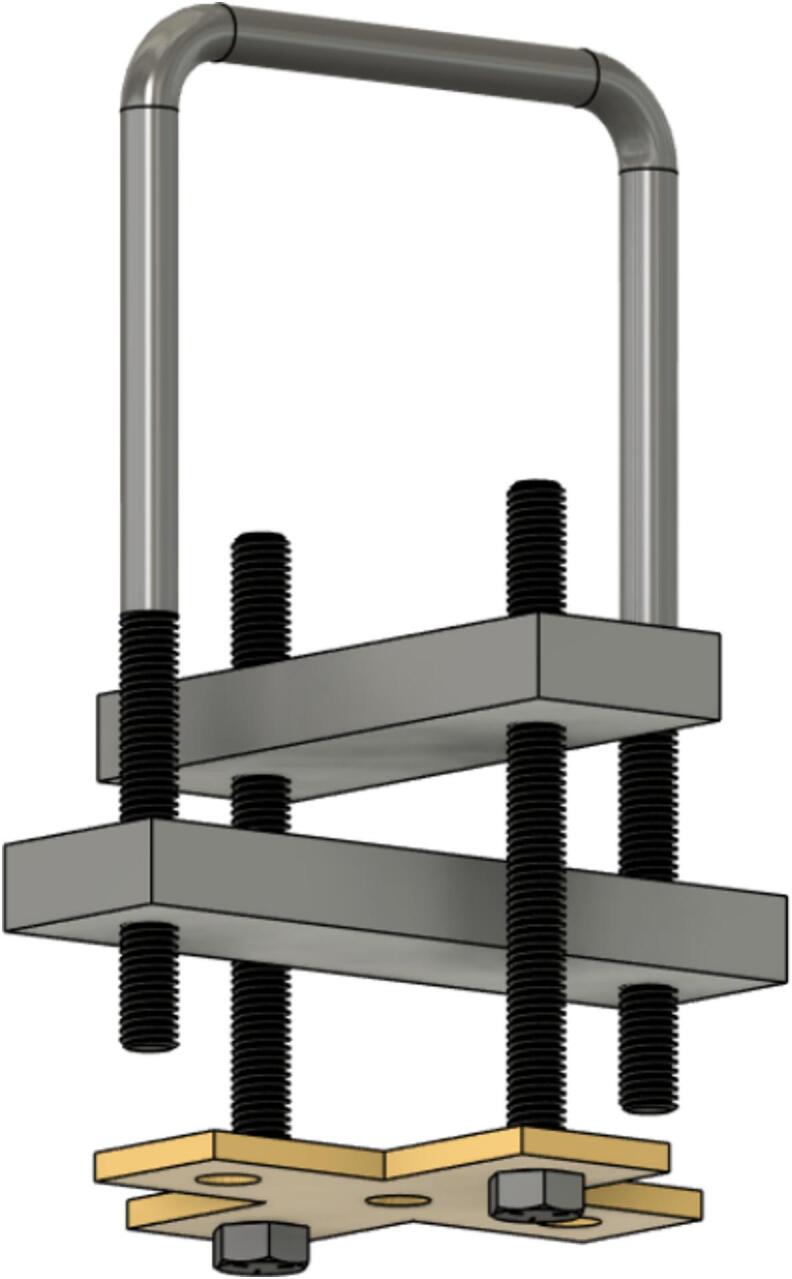
The location of through holes in the 17.78 cm (7-inch) and 15.24 cm (6-inch) pieces cut from ID49 are shown relative to the U-bolt (ID 30) and the Strut Channel Cross Bracket (ID 24).

Part ID49, the 13-inch (33.02 cm) steel bar, 1.905 cm (¾″) thick and 5.08 cm (2″) wide, should be cut into 15.24 cm (6″) and 17.78 cm (7″) pieces. Each should have two through holes drilled using a ½-inch (1.27 cm) bit. Fig. 4 illustrates how the hole positions can be marked using a U-bolt (ID30) for the 17.78 cm (7-inch) piece and a Strut Channel Cross Bracket (ID24) for the 15.24 cm (6-inch) piece.

The 2-foot (60.96 cm) aluminum part (ID40) should be cut into two 9.398 cm (3.7″) and two 20.828 cm (8.2″) pieces. They will be drilled and tapped as needed during assembly to secure acrylic sheets.

Acrylic plates should be sorted by color and cut, using the patterns provided as design files, using a laser cutter with a minimum cutting bed dimension of 45.72 cm × 53.34 cm (18″ × 21″).

### 5.2 Assembly of the structural frame, force application section

#### 5.2.1 Frame sub-assemblies

**Fig. 5:**
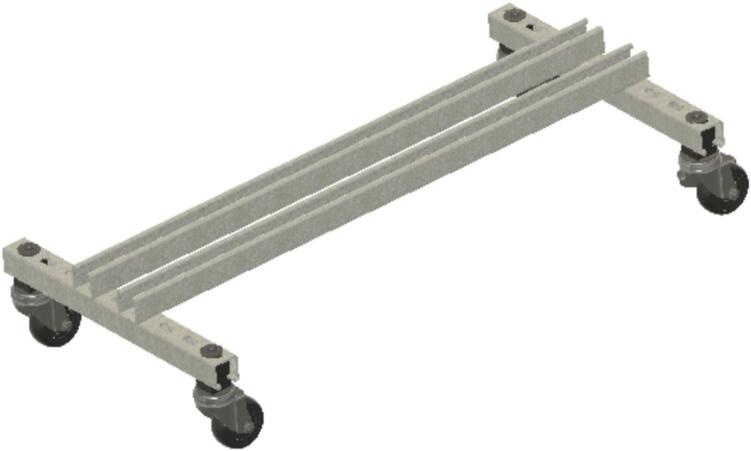
Sub-assembly SA-A.

Build sub-assembly SA-A, shown in Fig. 5, by connecting the lower struts (ID3) and caster wheels (ID20 and ID21) to ID4 using channel bolts (ID6) and nuts (ID34).

**Fig. 6:**
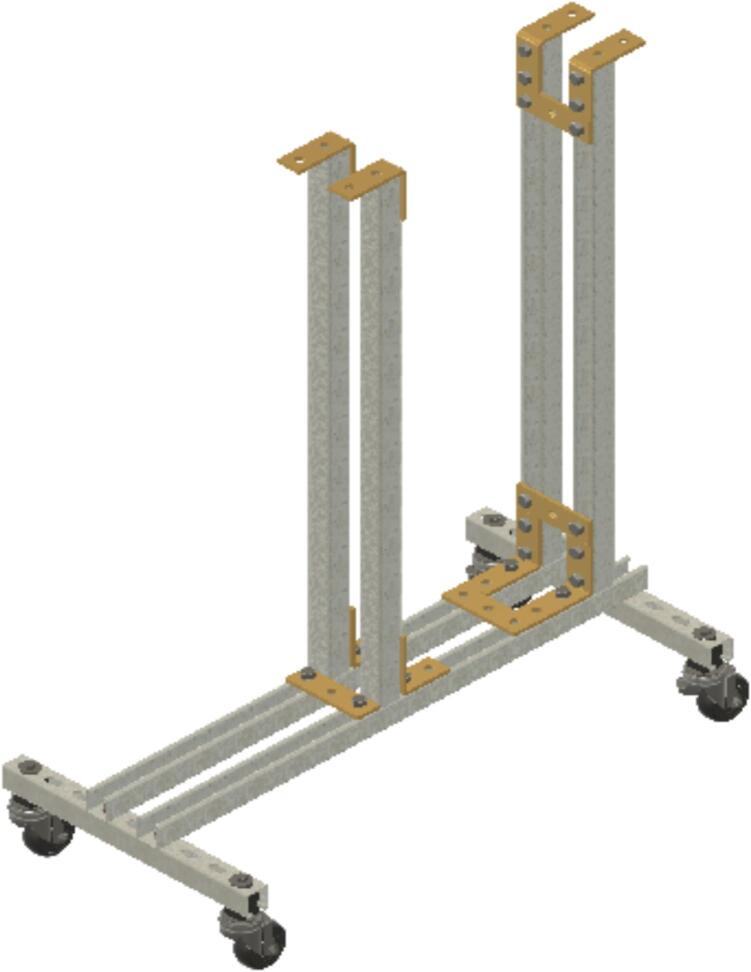
Sub-assembly SA-B.

Install ID1 by bolting ID18 and ID19 brackets on at the top of ID3. Then install two other ID18 brackets at the top of the ID1 struts to make SA-B as in Fig. 6.

**Fig. 7:**
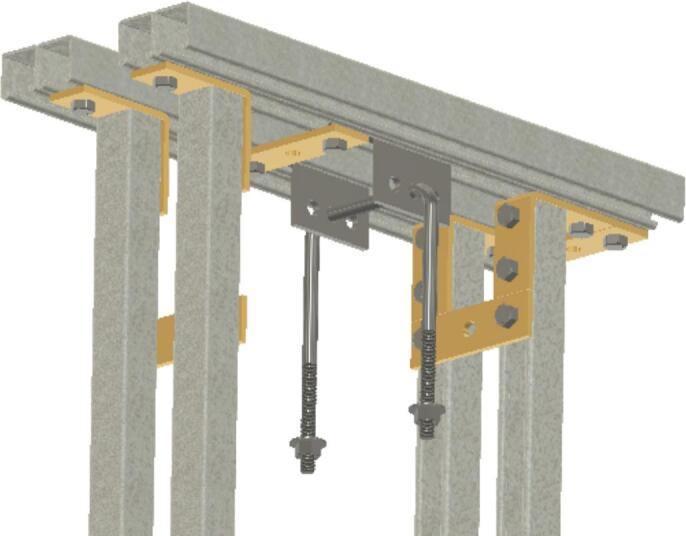
Sub-assembly SA-C.

Insert U-bolt (ID30) into the two trolleys (ID15), insert them into the Side-to-Side strut channel (ID36) and install ID36 into ID1 with ID18 brackets. Then install ID19 brackets to create SA-C, as shown in Fig. 7.

**Fig. 8:**
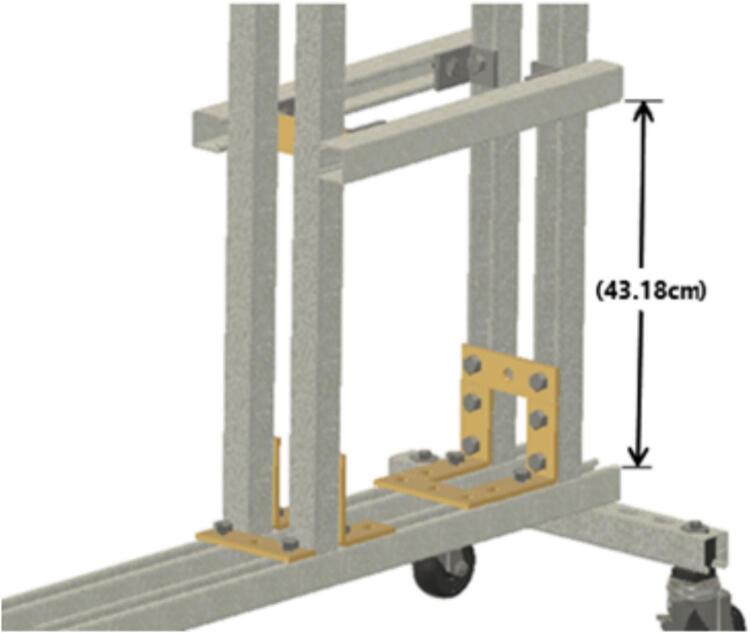
Sub-assembly SA-D.

Install the ID2 strut channel using ID39 brackets to ID1 at a position 43.18 cm (17 in.) from the top of ID3) to create SA-D, Fig. 8.

**Fig. 9:**
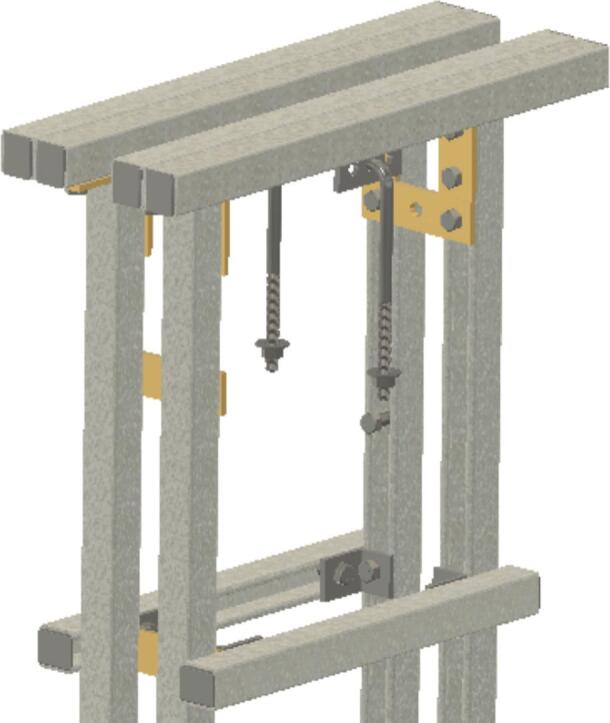
Sub-assembly SA-E.

Install 1D37 caps into the exposed ends of the strut channel parts to create SA-E, like in Fig. 9.

**Fig. 10:**
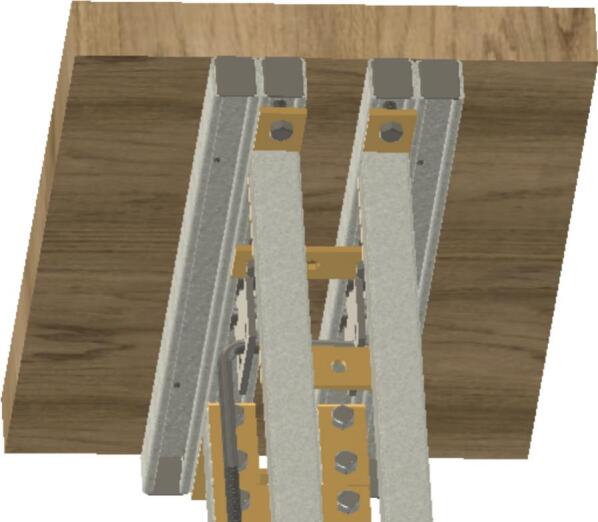
Wood cutting board installation.

To install the wood cutting board (ID7), which serves as the “roof” stabilizing the test rig (see Fig. 10), drill holes in the ID36 Side-to-Side strut channel and secure it with brass screws (ID45).

#### 5.2.2 Force application system sub-assemblies

**Fig. 11:**
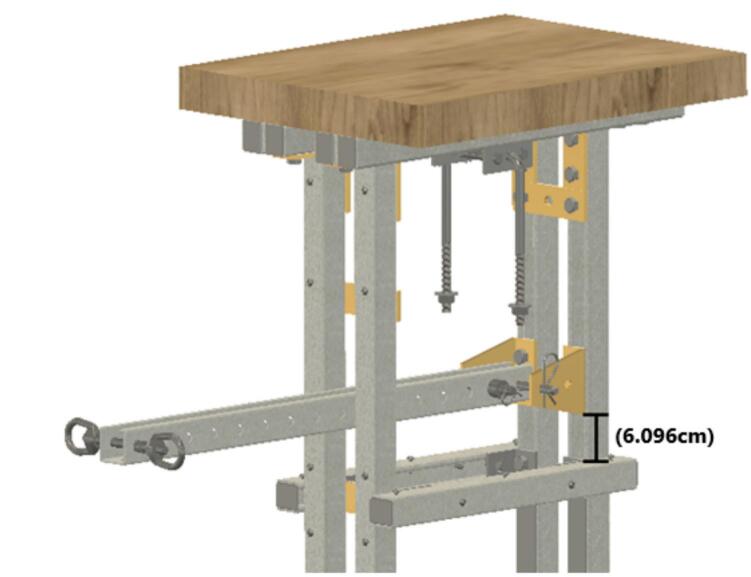
Telescoping strut installation.

Attach the ID16 brackets to ID1, 6.096 cm (2.4 in.) above ID2, then install the telescoping strut (ID17) using the ID23 clevis pin to secure it in place. There will be a substantial gap between the telescoping strut (ID17) and the ID16 brackets. The gap ensures that there is no contact friction between the bracket (ID16) and the lever arm (ID17). At the end of the lever arm use an ID33 threaded rod, ID34 nuts, and ID32 eye nuts to create the attachment point for the weights. See Fig. 11.

### 5.3 Installation of sample fixture components and measurement equipment

#### 5.3.1 Sample fixture sub-assembly

**Fig. 12:**
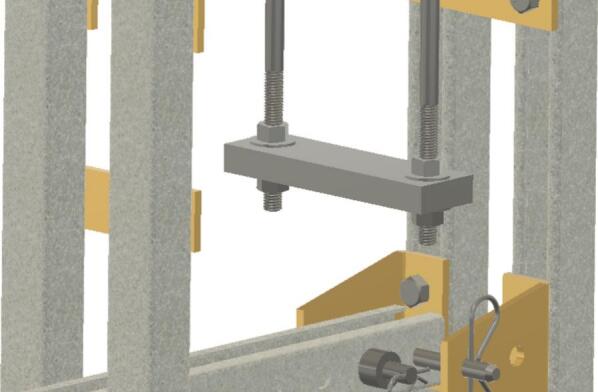
7-inch (17.78 cm) steel piece attachment.

Attach the 17.78 (7-inch) steel piece (cut from ID49 in Section 5.1.2 above), previously drilled, to the hanging U-bolt (ID30) using washers (ID42) and nuts (ID34). See Fig. 12.

**Fig. 13:**
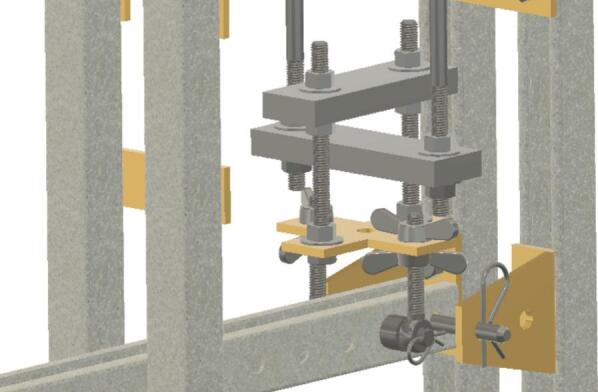
Sub-assembly SA-F.

To create sub-assembly SA-F, first, attach the two ID31 components to the lever arm using the clevis (ID25) and cotter pin ID48, ensuring the ID22 spacers hold them securely. Mount the ID24 bracket with four ID41 wing nuts. Then use two 6-inch-long (15.24 cm) screws (ID26), along with nuts and washers to connect the 15.24 cm (6-inch) piece to ID24. See Fig. 13.

**Fig. 14:**
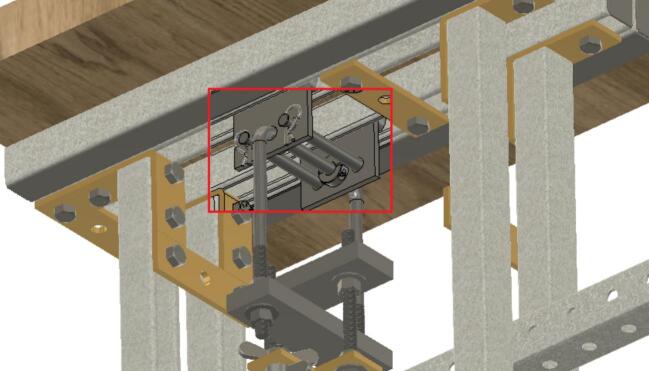
Clevis and cotter pins inserted through the trolleys and collars added to the U-Bolt.

To better maintain alignment between the trolleys, two ID 25 clevis pins are placed in the unused holes in the trolleys and secured with cotter pins ID48. Two ID43 collars are attached to the U-Bolt. See Fig. 14.

#### 5.3.2 Measurement equipment Sub-assembly

**Fig. 15:**
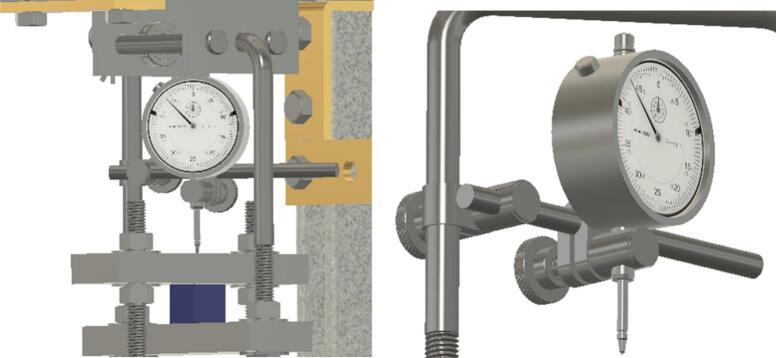
Sub-assembly SA-G. SA-G is referred to as the sample fixture assembly and sub assembly SA-F (Fig. 13) is included in it.

Install and secure the displacement sensor (dial indicator ID8) to the U-bolt (ID30) with the connectors (ID27, ID28, ID29) to build sub-assembly SA-G, shown in Fig. 15.

### 5.4 Build sample enclosure

To keep the sample isolated from the surrounding environment, acrylic sheets are used to enclose the sample compartment, and gaps within the strut channel are closed off using strut channel cover (ID38) and ID37 caps. The enclosure is not air-tight, but it is largely sealed, including sliding acrylic plates above and below the lever arm that move up and down with it. The basic design is intended to operate at the same temperature and humidity as the surrounding environment, but it could be modified to include temperature and humidity regulation within the enclosure.

#### 5.4.1 Attach the acrylic sheets

The acrylic sheets (ID11, ID12, ID13) should be cut using a laser cutter. The cut panels include the 18″ × 21″ (45.72 cm × 53.34 cm) clear left and right-side panels, the clear back panel, two clear sliders, a red bottom panel, red front panel, red internal panel, and a red lower front panel and lower back panel.

Laser-cut holes in the panels can be used as a guide to mark all the locations where the panels need to be screwed onto the frame. Once the locations are marked, drill holes in the strut channel using a 3/16-inch (0.47625 cm) bit.

Tap the drilled holes using a 7/32-inch (0.555625 cm) tap and tapping oil. At the top of the two acrylic side panels, there are four holes: two holes are for ID 44 threaded studs, and the other two are for machine screws (ID46). With ID 46 machine screws attach the red acrylic front panel, the red acrylic lower front panel, the red acrylic bottom panel, and the clear acrylic back panel. The threaded studs (ID44) hold the large side panels while the machine screws are attached or removed. They make it easier to remove the side panels for access to the sample chamber and make it easier to reattach the panels afterward.

**Fig. 16:**
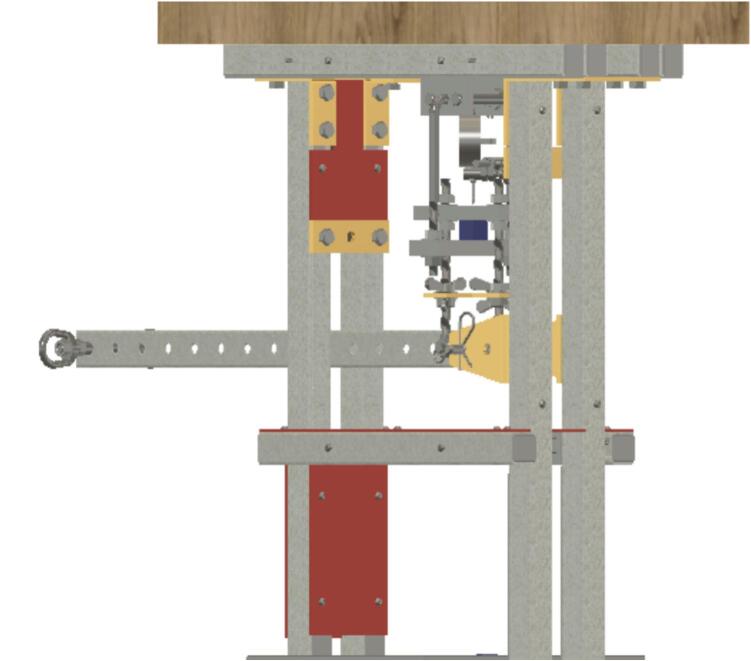
Two red sheets to be installed onto aluminum parts. (For interpretation of the references to color in this figure legend, the reader is referred to the web version of this article.)

The red internal panel and the lower back panel shown in Fig. 16 need four aluminum parts that were cut from ID40 as described in 5.1.3. Using the previously described method, mark the drilling locations on the four aluminum parts based on the holes in the acrylic sheets, then drill and tap the holes. The aluminum parts should be held in place in the strut channel by hand, then, the red sheets can be installed by securing them with ID46 screws.

After installing the red acrylic panels, there will be open spaces above and below the lever arm, which will be covered by movable clear acrylic slider panels. To properly position these movable panels and ensure they move in the correct direction, slider guide foams (ID14) should be used. Cut the foam to length with the bottom foam pieces being 22.86 and 25.4 cm (9 and 10 in.) and the top foams being 21.59 and 19.05 cm (8.5 and 7.5 in.), respectively. Similarly, for both the top and bottom parts, start by positioning the appropriate acrylic sheet between the two pieces of cut foam. Then, insert all three components into the gap between the struts simultaneously, ensuring that the smaller foam pieces are oriented outward in both the top and bottom gaps. Finally, attach the installed sheets to the lever arm via a zip-tie, piece of wire, or string.

#### 5.4.2 Seal strut channels

Cut the strut channel covers (ID38) to size and use them to seal all the open strut channels.

### 5.5 Auxiliary parts

Attach the weight storage basket (ID9) to the lower part of the frame with bolts and nuts.

### 5.6 Design alternatives

#### 5.6.1 Different types of displacement sensors

To enhance data accuracy and simplify data collection, digital sensors can be used in place of analog dial indicators. These sensors can automatically store and transmit data to computers. Additionally, a highly sensitive Nano-Gauge (Mad City Labs, Madison, WI), can be used to achieve 1.524 nm or 0.00000006 in. resolution (compared to the typical 3810 nm (0.00015 in.) accuracy of analog gauges).

#### 5.6.2 Uninterruptible power supply (UPS)

If electronic sensors are added, there is room for an uninterruptible power supply (UPS) to be mounted vertically on the rear vertical struts (ID1) of the frame. The UPS will prevent data loss during power outages while the digital gauges collect data.

#### 5.6.3 DIN rail electronics

The front portion of the sample chamber can be used to house electronics such as temperature controllers, chamber heaters, etc. A DIN rail can be attached to the red acrylic internal panel by creating holes in it and drilling and tapping the aluminum plate behind it. Power wiring can be routed under the butcherblock wood top, in the gap between the two side-by-side channel struts ID36, then out the rear of the test rig so that the power wiring can be kept clear of the lever arm and fed to the UPS if added.

#### 5.6.4 Load sensor

To enhance the rig, a load measurement sensor can be added to ensure the stability of the applied force. This sensor requires no additional equipment if it has a maximum diameter of 7.0108 cm (2.76 in.) and a height of 3.9878 cm 1.57 in. (1.57 in.); simply place it on the lower plate in the sample fixture assembly, SA-G, where the sample would normally go and place the sample on top of the load cell.

#### 5.6.5 Automatic temperature and humidity recording

A temperature and humidity sensor can be added, and it will be convenient if they are automated to log and transfer data to a computer at predetermined intervals.

## 6 Operation instructions

A single person can effectively handle the entire testing process, but having two is recommended.

### 6.1 Loading the sample

To initiate the test, unscrew the securing screws to remove the clear acrylic side panel from one side (the side that allows for gauge readings).

**Fig. 17:**
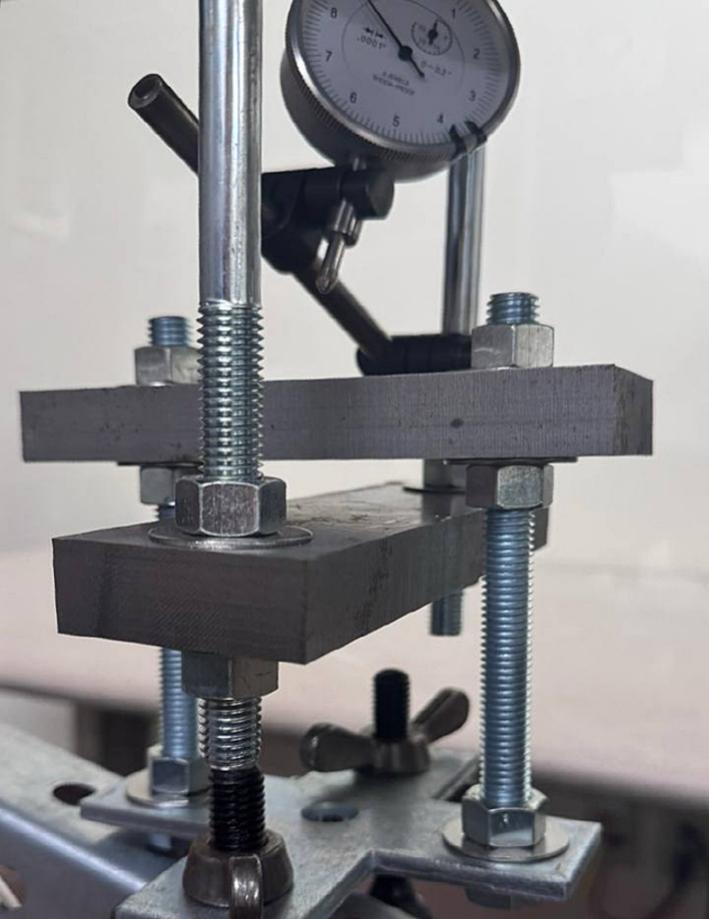
Raising the upper fixture plate of SA-G to position the sample between the upper and lower fixture plates.

Begin by raising the creep sensor along with the upper plate of the SA-G sub-assembly, slightly to create enough room for positioning the sample between the fixture plates (see Fig. 17 for reference).

**Fig. 18:**
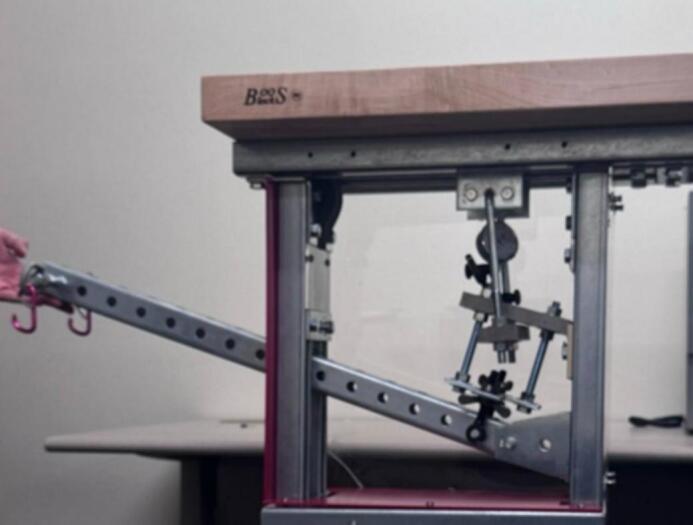
Lifting the lever arm to make space in the sample fixture assembly SA-G for sample loading.

Lift the lever arm high enough to ensure there is more space between the upper and lower fixture plates (ID49 from Section 5.1.2) than the height of the sample, see Fig. 18.

After centering the sample between the fixture plates (from Section 5.1.2), ensure the lever arm stays centered between the ID16 brackets, and then gently lower it.

The angle of compression applied through each load-transmitting component influences the load experienced by the sample. It is crucial to ensure that the entire sample fixture assembly (SA-G) is aligned vertically so that it is parallel to the direction of gravity acting on the shot bags.

It may be necessary to repeat this step several times to ensure that the sample is centered within the sample fixture assembly and that all vertical elements of the sample fixture assembly are aligned. The use of a 90-degree square and plumb line is recommended to assist in this step.

Before continuing, ensure that the sample fixture assembly is aligned, and verify that the lever arm is centered between the ID16 brackets and the two upright beams (ID1) at the front of the rig through which it passes.

This step takes some patience and practice, but if alignment proves difficult, check to make sure that brackets ID16 are square and that the rest of the creep test rig is also square.

**Fig. 19:**
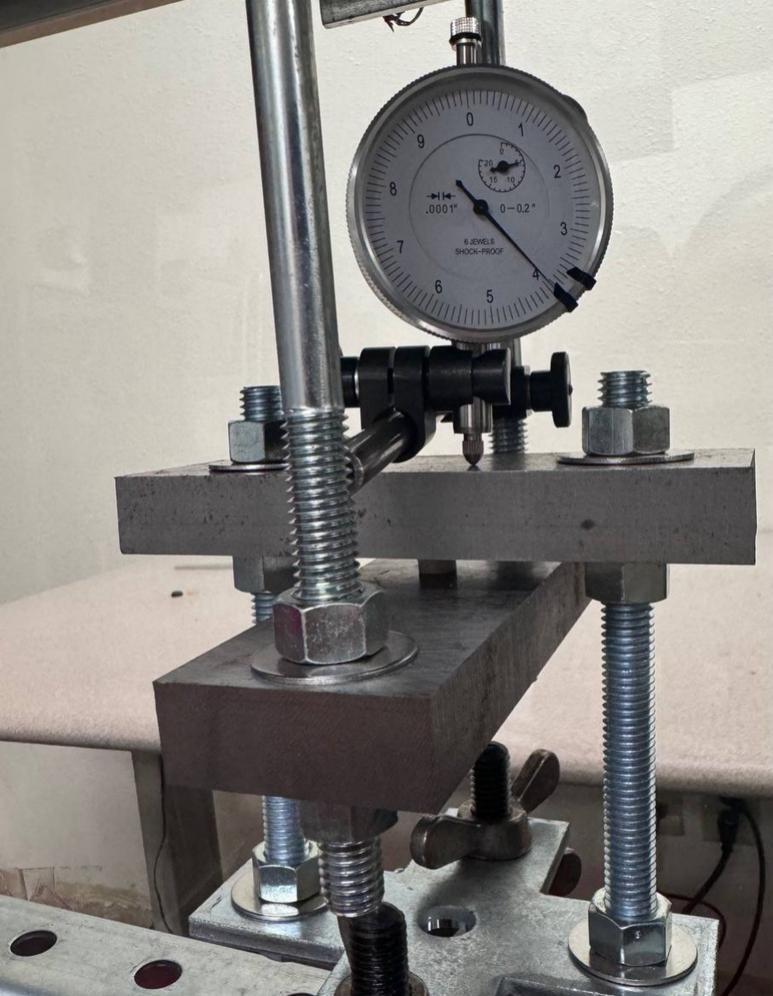
Dial indicator with plunger perpendicular to the upper fixture plate.

Lower the dial indicator sensor ID8 so it touches the upper plate of the sample fixture assembly component perpendicularly. This arrangement allows the dial indicator’s plunger rod to move freely and perpendicular to the sample's surface. Make sure the plunger rod has enough space to move to track creep data. See Fig. 19.

(Optional but recommended) Place an operating temperature and humidity sensor inside the chamber, ensuring the display is easily visible.

Reattach the acrylic side panel to fully enclose the testing environment. Keeping the panel on during runs is recommended to prevent bystanders from reaching inside and potentially being exposed to pinch points.

Before starting the loading phase, note the initial creep gauge reading along with the time as the first recording.

Gently hang the necessary weights onto the ID32 Eye nuts using s-hooks, carabiners, or a similar attachment method. Apply the weights at a steady rate and avoid dropping them onto the arm or other motions that would result in a sudden force impulse on the arm.

### 6.2 Collecting displacement data

As the sample compresses, the upper fixture plate moves downward relative to the dial indicator, and the amount of compression can be monitored by logging the dial indicator readings and the times at which the readings were taken. It is convenient to automate this process, and several options were discussed in Section 5.6.1, but the default build requires manual data logging. Runs may last for multiple days, weeks, or even months, so it is not practical to collect data at specific time intervals, and for most applications, it is not necessary. This instrument is intended for measuring second-stage creep, which is primarily due to viscous deformation, where the creep rate will be nearly constant. Relatively large time gaps between measurements should be acceptable. In first-stage creep, the motion is due to viscoelastic compression, and the creep rate changes relatively quickly. If first-stage creep is of interest, more frequent data collection will be needed in the first few days of the run.

Creep data is often analyzed by fitting the experimental data to a model, such as the power law model,

(1) *x* = *At^n^*

where *x* is travel (measured with the dial indicator ID8), the uniaxial displacement of the top plate, which represents the compression of the sample, *A* and *n* are constants associated with the sample material and environmental conditions, and t is time. Various methods can be used to fit the power law equation to the sample data, but the Solver tool in Excel is an option available to many people due to the widespread use of Microsoft products. Once the values of *A* and *n* that result in the best fit of the power law model to the experimental data are determined, the equation can be used to predict travel at any arbitrary time. Other creep models can also be used to fit the data [[11], [12], [13]]. In equation 1, we represent creep as travel because it directly relates to the measurement taken with the dial indicator. It can also be represented as strain, *∊*, where *∊* = −*x*/*L* and *L* is the initial height of the sample. The power law model covers first and second-stage creep but if only second-stage creep is considered, it should be possible to use linear regression to fit the data as a straight line.

### 6.3 Device operation limitations

It is recommended to refer to the standard [10] for sample dimensions; however, the space between the ID49 steel components allows for testing samples with a maximum height of up to 5.08 cm (2 in.). The sample diameter or width can be up to 5.08 cm (2 in.), but 2.54 cm (1 in.) is the recommended maximum. There is no minimum height, but the rig is not intended for testing thin films. Likewise, there is no minimum diameter or width, but samples less than 0.635 cm (1/4 in.) may be difficult to load.

Although the creep test rig’s frame enables the application of significant forces, it is recommended to avoid placing a weight totaling more than 45 kg (100 lb) on the lever arm.

Care should be taken so that the creep test rig is not bumped, jostled, or subject to vibrations during the test, otherwise, the results could be unreliable.

## 7 Validation and characterization

**Fig. 20:**
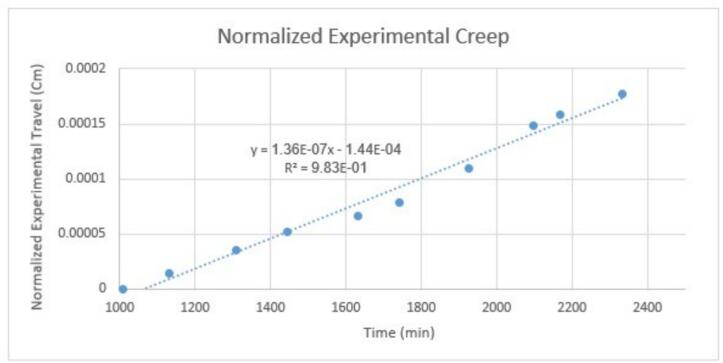
Normalized experimental data on 1000 min − the regression line has a slope of 1.36 × 10^−7^ cm/min with R^2^ of 0.983.

The initial conditions of the test, such as the magnitude of the initial force applied when placing weights on the lever, impact the overall test results by either consistently increasing or decreasing all the creep data by a relatively constant amount. The creep rate in the second-stage creep region is not changed, but the creep curve may be offset vertically up or down. For this reason, it is advised that the gathered data be normalized to a specific time point (as shown in Fig. 20). This involves subtracting the travel (*i.e.* the displacement measured by the dial indicator, ID8) at time *t_norm_* from the travel values at all subsequent times. The time *t_norm_* should be near the start of the second-stage creep region, but the specific value will depend on the sample and applied load; for LDPE, the authors have found that *t_norm_* = 1000 min works well.

**Fig. 21:**
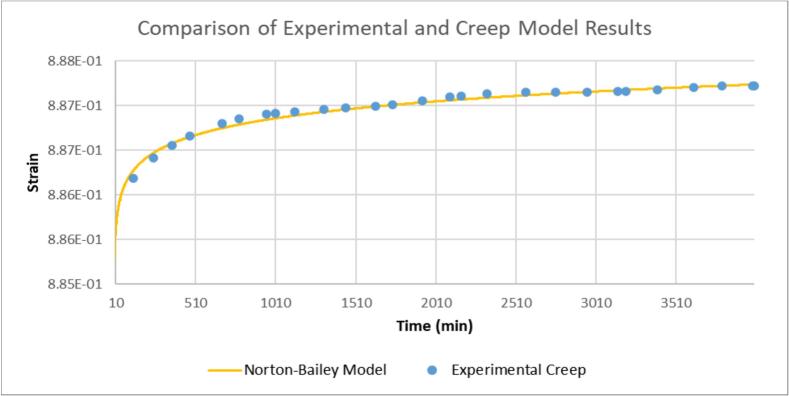
Comparison of experimental and creep modeling results using a Norton-Bailey/power law fit.

To validate the data from the test rig, we examined the Norton-Bailey model [14], one of the well-known models for first and second-stage creep prediction which demonstrates that the experimental data collected from the test rig—when normalized at a specific time, such as 10 min in Fig. 21—fits well with this model.

(2) *∊* = *Bσ^m^t^n^*

In this model, *∊* is strain, *B*, *m*, and *n* are constants, *σ* represents stress, and t denotes time. To simplify the Norton-Bailey model into a power law, A/L = Bσ^m^, which yields the equation 1 power law [15] discussed in Section 6.2. Fig. 21 is a graph of experimental creep data compared with a power law fit, with *A* = 0.278727145 and *n* = 0.00031; the values of *A* and *n* were determined via fit optimization.

### 7.1 Effects of contact friction

**Fig. 22:**
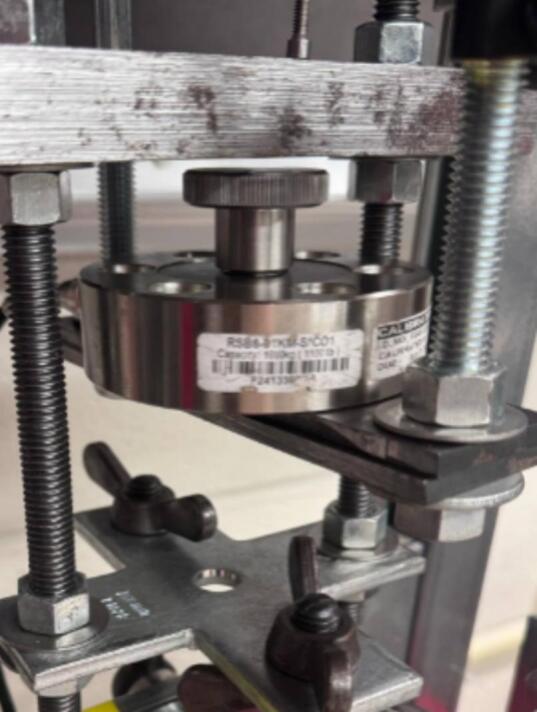
Load cell positioned in the sample fixture assembly.

**Fig. 23:**
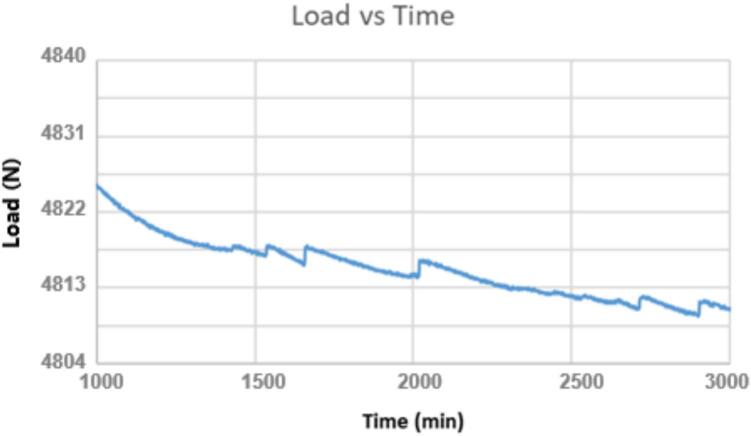
Load cell data showing the effects of contact friction on the force applied to the sample.

In the early creep test rig design, ID16 brackets were rotated 180 degrees so that there was a very small gap between the lever arm ID17 and the brackets. In some cases, the brackets were pressed against the lever arm, resulting in contact friction. To determine the force being applied to the sample, a load cell (Fig. 22) was placed in the sample fixture assembly, and a run was done with Brackets ID16 flipped so that they contacted the lever arm ID17. The contact friction created a saw-tooth pattern where the peaks in Fig. 23 represent slippage such that contact was briefly lost and contact friction did not counter the applied load, followed by a decrease in load experienced by the sample as contact friction recovered.

**Fig. 24:**
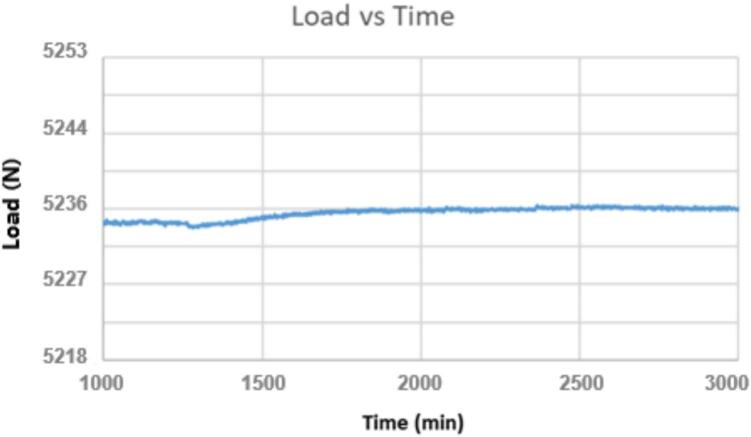
Load cell data from a normal run without contact friction. There is a change in load of a little less than half of a pound from 1000 min to 1700 min, but this small change is 0.042 % of the total load and is considered acceptable.

In addition to the spikes shown in Fig. 23, contact friction also causes drift on the applied load over the course of the run. The configuration of the ID16 brackets means that care must be taken when starting a run to ensure everything is square, but it ensures contact friction will not interfere with the experiment. Fig. 24 shows the load cell data for a normal run with a wide gap between brackets ID16 and lever arm ID17. It is important to note that, to prevent any unintended friction between the lever arm and the other nearby components, each test must be conducted with careful attention to ensure the lever arm does not touch the ID1 struts as well.

### 7.2 Self-leveling mechanism

**Fig. 25:**
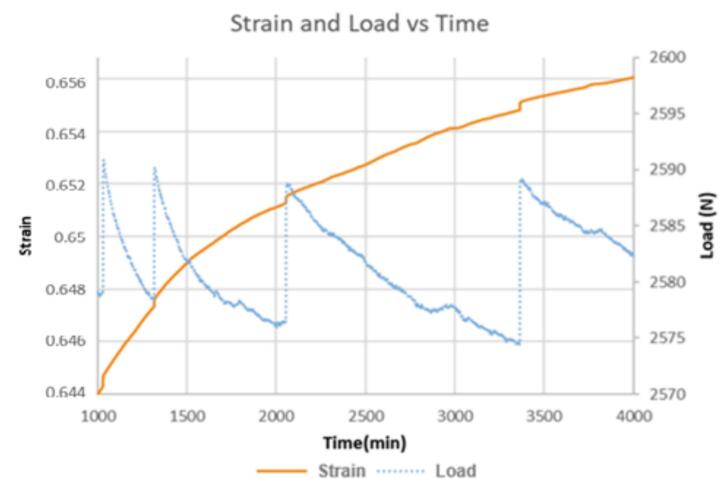
Load vs travel data with the trolleys fixed in place so that the sample fixture assembly must tilt when the lever arm moves. The ID16 brackets are intentionally pressed against the lever arm ID 17 to create contact friction and load spikes. Jumps in the travel curve that align with load spikes are due to the shear deformation of the sample. Travel data was collected with a Nano-gauge which logs high-precision data every minute.

**Fig. 26:**
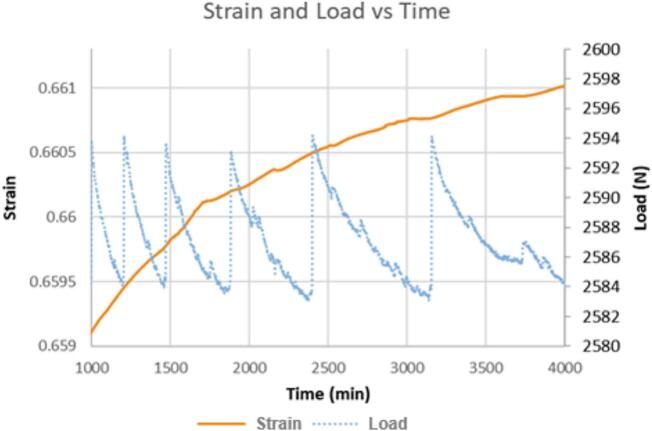
Load vs travel data with the trolleys free to move so that the sample fixture assembly remains vertical as the lever arm moves. The ID16 brackets are intentionally pressed against the lever arm ID 17 to create contact friction and load spikes. No jumps in travel corresponding to load spikes are observed because shear forces are prevented by the trolly self-leveling mechanism. Travel data was collected with a Nano-gauge that logs high-precision data every minute.

The self-leveling mechanism ensures that the sample fixture assembly remains vertical throughout the entire run. To understand the role it plays, the trolleys (ID15) were fixed in place by bolting (ID19) straight brackets in front of and behind them. The ID16 brackets were then reversed so that they would contact the level arm (ID17) and intentionally create load spikes as seen in Fig. 23 for this demonstration. Comparing the strain and load data, jumps in strain can be observed corresponding to the load spikes in Fig. 25. In contrast, when the ID 19 brackets were removed and the trolleys were free to move, there were no jumps in strain corresponding to load spikes, see Fig. 26.

**Fig. 27:**
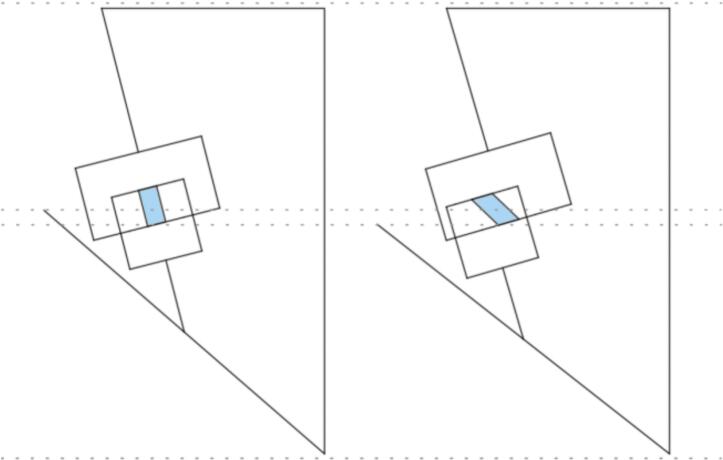
Shear compression. The diagrams represent the creep test rig with the trolleys prevented from moving causing the sample fixture assembly to tilt. The blue-shaded regions represent the sample, and the diagrams show how shear transforms the sample from a rectangle to a parallelogram. The shaded area is the same in both diagrams, but the parallelogram results in a reduction in height which lengthens the sample fixture assembly. The lever arm can, therefore, move downward due to sample shear as well as sample compression. If the sample fixture assembly is tilted as depicted in the diagrams, then the sample will experience a shear force resulting in shear deformation. With the trolleys serving as a self-leveling mechanism the sample fixture assembly remains vertical over the entire run and the sample will not experience shear forces, assuming the sample was properly aligned at the start of the run. (For interpretation of the references to color in this figure legend, the reader is referred to the web version of this article.)

To explain the behavior in Fig. 25, Fig. 26, consider that the downward motion of the lever arm occurs due to compression of the sample, which causes lengthening of the sample fixture assembly. This lengthening allows the lever arm to move. When the trolleys are pinned and cannot move, compression of the sample results in both downward motion of the lever arm and tilting of the sample fixture assembly. As a result of the tilt, a shear force is created and causes shear deformation of the sample. Because the volume of the sample stays approximately the same, the shear deformation results in the height of the sample decreasing (*i.e.* compression) due to the sample shifting sideways, see Fig. 27. The dial indicator (ID 8) cannot distinguish between compression due to shearing sideways vs uniaxial compression, but it requires less force to shear the sample, so that the load spikes create jumps in the strain vs time graph, and generally results in faster-perceived creep.

It is important to note that in normal operation, there should be no contact friction on the lever arm, and no load spikes should occur. These spikes have been intentionally created here to induce sudden shear when the trolley is pinned in order to demonstrate the utility of the trolleys. It is important to note that even if load spikes were not present (*i.e.* bracket ID16 was in the correct orientation), shear would still occur if the trolley were pinned or not included as part of the test rig. The shear would not show up as dramatic jumps in strain but would result in an erroneously higher creep rate measurement.

**Fig. 28:**
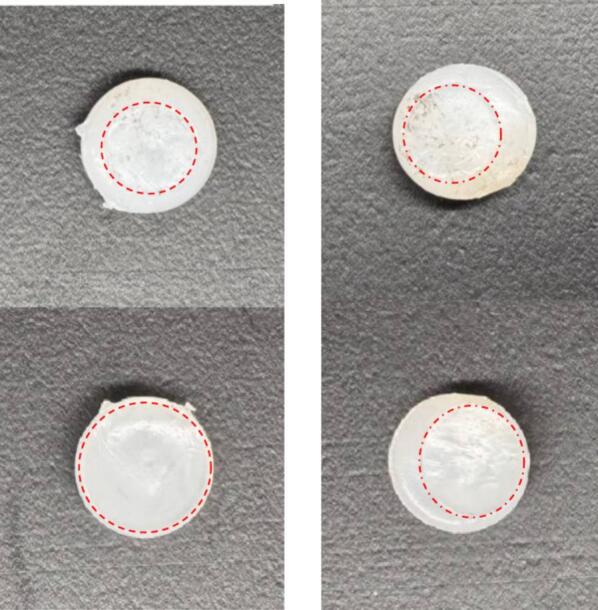
Comparison of two samples post-run. The photos on the left show the top and bottom of a sample that had no shear force. The photos on the right show a sample that did have sear force.

Even with the trolleys working, it is still important that the sample should be centered, and the sample fixture assembly squared so that the load passing through the sample always remains vertical, passing through the sample axially. Fig. 28 compares a sample that was compressed uniformly vs one that had shear. After removing the sample from the rig, it will swell over the course of minutes as the stored potential energy from elastic deformation is released. The sample should be observed during this time to determine if the impression marks from the plates that were holding it are centered or offset. If they are offset, it indicates that there were shear forces during the run and that the data may be suspect. Shear can occur if the sample was not centered on the fixture plates or if the sample fixture assembly SA-G was not square.

### 7.3 Validation

**Fig. 29:**
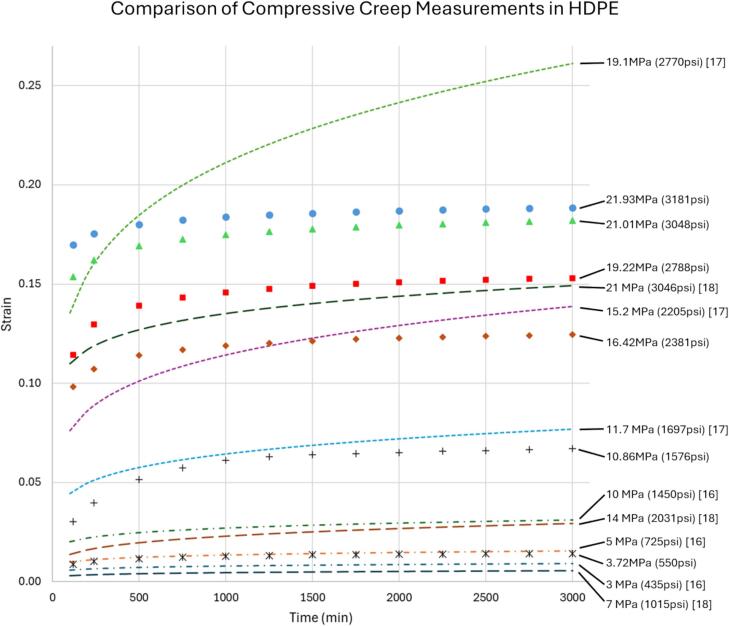
Comparison of compressive creep curves for HDPE measured by other authors and with the rig described here. Lines represent the work of other authors and are power law fits (R^2^ >= 0.96) of their published data. A custom rig was used for some of the fits (long dash lines) [18], and the others were collected on two different commercial systems (dash-dot) [16] and (short dash) [17]. Data points on the graph represent actual data collected with the rig described here.

We compared creep curves of high-density polyethylene (HDPE) generated with the rig described here to curves generated by other authors. We used DigitizeIt (digitizeit.xyz, Braunschweig, Germany) on graphs in three journal articles [16,17], and [18] to get strain vs time data points, which we used to create the power law fits (see Section 6.2), which are shown in Fig. 29. Elleuch and Taktak [16] used a commercial tensile testing machine (Lloyd-instruments) to measure compressive creep under stress of 3 MPa (435 psi), 5 MPa (725 psi), and 10 MPa (1450 psi). The power law fit for these three curves were excellent, all had an R^2^ coefficient of determination at 0.994 and above. Zhang and Moore [17] used a commercial servo-hydraulic test rig (MTS Systems Corp) to measure compressive creep under stress of 11.7 MPa (1697 psi), 15.2 MPa (2205 psi), and 19.1 MPa (2770 psi). The power law fit for these three curves were excellent, all had an R^2^ coefficient of determination at 0.998 and above. Jeya and Bouzid [18] used a custom-made creep rig that employed hydraulics to generate compressive load. They measured compressive creep at 7 MPa (1015 psi), 14 MPa (2031 psi), and 21 MPa (3046 psi). The power law fit for these three curves were very good, all had an R^2^ coefficient of determination at 0.96 and above. We performed creep tests on our rig using 3/8-inch (0.9525 cm) diameter HDPE rod made to ASTM D4976 and UL 94HB standards (McMaster-Carr, 8624 K12), cut to a height of 0.823 cm (0.324 in.). Tests were performed at 3.72 MPa (550 psi), 10.86 MPa (1576 psi), 16.42 MPa (2381 psi), 19.22 MPa (2788 psi), 21.01 MPa (3048 psi), and 21.93 MPa (3181 psi).

In Fig. 29, comparing results between authors, there are discrepancies in the overall magnitude of strain, such that some curves taken at higher stress by one author are beneath curves taken at lower stress by another author. Likewise, the slopes of the curves in the linear region (generally the region after 1000 min) differ between authors. These discrepancies are likely due to differences in the HDPE samples used. While the name HDPE represents the chemical structure and degree of branching of the polymer, there is still a great deal of variability between HDPE products from different suppliers. Temperature may also play a role, but our data and the data from the other authors were collected at stable ambient temperatures [[16], [17], [18]], so differences in the HDPE material are more likely the cause.

While there are some discrepancies in Fig. 29, the results indicate that the rig described here performs similarly to other rigs. Better validation of our rig could be achieved with a side-by-side comparison utilizing the same sample material and at the same temperature, but the authors do not have access to other commercial or home-built rigs for such a comparison. An additional challenge is the variability within thermoplastic samples themselves, which makes it difficult to have a true standard sample for comparison.

When conducting multiple tests on samples of the same dimensions, processed the same way, and made from the same material, we see differences in the shape of the creep curve, but this is not caused by the rig, it is due to variability in the sample material itself. For this reason, the variability and precision of the rig cannot easily be evaluated by measuring samples but must instead be considered by other means. There are four elements to consider: the precision of the stress, the stability of the applied load, and the precision of the travel and time measurements, and the accuracy of the sample height for calculating strain.

To calculate stress, the cross-sectional area of the sample must be known, and this is limited by the fact that the cross-section changes during compression. The nominal stress associated with the run is an estimate of the true stress which changes as the sample compresses. As a result, the reported stress is approximate because there is no mechanism for measuring the diameter of the sample during the run. This situation is common to most creep test rigs, not unique to ours. While the stress may be approximate, as long as the load is constant, the reading for the rate of compression of the sample will be accurate. In the previous sections, we have described how we eliminated major sources of error, such as contact friction and unintended shear, that could cause the applied load to be different than expected or to change during the run. We believe that the applied load will be stable when the rig is used properly.

If the load is stable, the precision of the creep rate will depend only on the precision of the dial indicator reading and the device used to measure the time when the dial indicator reading was taken. Because creep is a slow process, it is sufficient to measure time in units of minutes, which makes nearly any time-measuring device acceptable. The main contributor to the precision of the rig is the precision of the dial indicator. The dial indicator measures compression of the sample directly, and so long as it is perpendicular to the upper plate that causes the sample compression, the only source of instrument error will be error within the dial indicator itself. As a result, the precision of the creep rate measurement should have the same number of significant figures as the dial indicator readings. The dial indicator used in this design (ID8) is specified by the manufacturer to measure changes in position to (0.0001″) with a precision of ±3.810 µm (0.00015″). Other displacement gauges can be used to achieve even higher precision.

It is common to report creep using strain, which is the travel measured with the dial indicator divided by the initial height of the sample. Error in determining the initial height of the sample and the initial zero value for travel can alter the strain measurements over the entire run. This is the most likely source of error for this creep test rig, but it is only a concern if the goal is to create a stress vs time graph. While this rig can be used to make stress vs time graphs, it was designed with the intention of measuring the creep rate by looking at the change in stress vs change in time. In this situation, errors in sample height and zero value of the dial indicator will cancel out and not be a factor. Measurement of creep rate precision will, as previously mentioned, be based on the dial indicator precision.

**Fig. 30:**
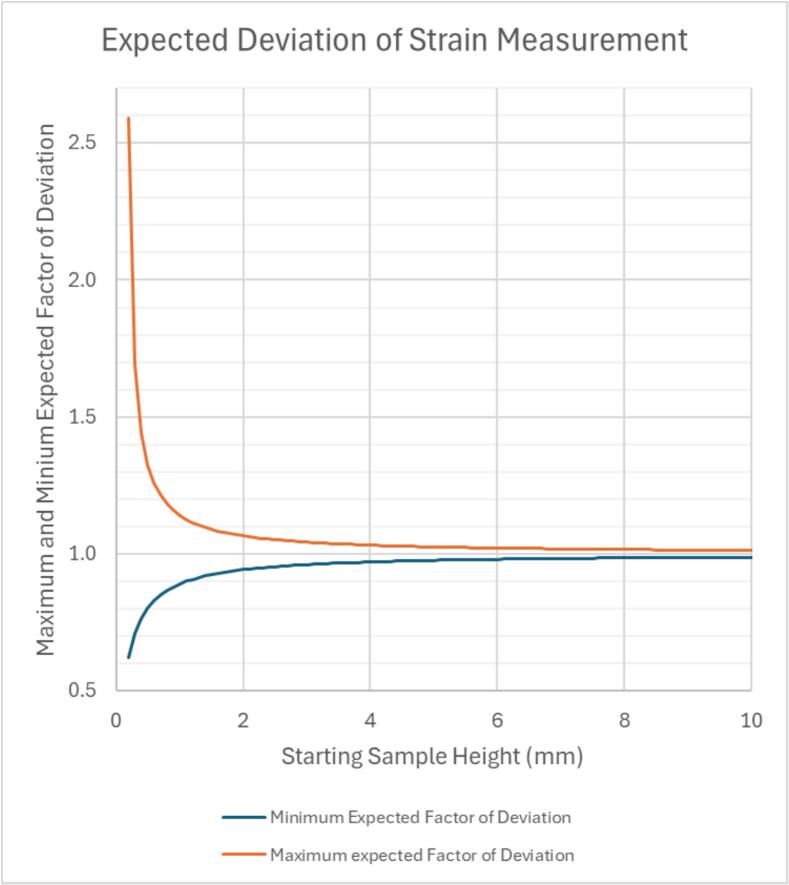
Expected deviation in the strain measurement due to sample loading. The graph is based on the tolerance interval for deviation from the true height of a steel block and shim gauge. It is intended to demonstrate the precision of the instrument itself, but actual measurement of thermoplastic samples will have greater deviation due to compression of the sample prior to getting the dial indicator in place.

Understanding the precision and repeatability for making strain vs time curves is complicated by the fact that thermoplastics are easily compressed and the load necessary to secure the sample in place (see Section 6.14) could cause compression prior to the dial indicator being put into place (6.15) such that zero travel on the dial indicator will underestimate the true travel since some compression already occurred. There is also an element of operator error in the initial setup if the sample is not square in the rig. While we could not measure the amount of thermoplastic compression that occurs prior to getting the dial indicator in place, we were able to look at the degree of operator error from an experienced user. Instead of using a thermoplastic sample, we used a steel spacer block and shim gauges of varying thickness to see how reproducible the height measurements were across multiple instances of loading and unloading the rig. We did 27 tests measuring the deviation of the measured value from what the true value should have been based on the shim gauge, and the standard deviation was 0.04214 mm. The tolerance interval containing 99 % of the population with 95 % confidence, based on a mean deviation of 0, gave a range of ±0.1228 mm. In other words, we should expect 99 % of the deviation observed to fall within these limits. Fig. 30 shows the minimum and maximum expected factor of deviation for strain based on sample height, where the factor of deviation should be multiplied by the true sample height to find the maximum and minimum measured height. The percent deviation is given by ABS(1-factor of deviation) × 100, such that the closer the factor of deviation is to 1 the more accurate the strain measurement. For sample height above 2 mm, which would be very small, the factor of deviation is already 1.065, which will not have an appreciable effect on the strain vs time curve. With that said, these measurements were done on a steel spacer block and shim gauge, which will not compress; they demonstrate that the instrument itself is precise, but for thermoplastic samples that will compress, the error will be larger. For this reason, graphs of strain vs time generated with this instrument should be considered approximate in terms of the strain. Measurements of creep rate, on the other hand, should be considered accurate with a precision dictated by the dial indicator used.

### 7.4 High-resolution creep data with the nano-gauge

**Fig. 31:**
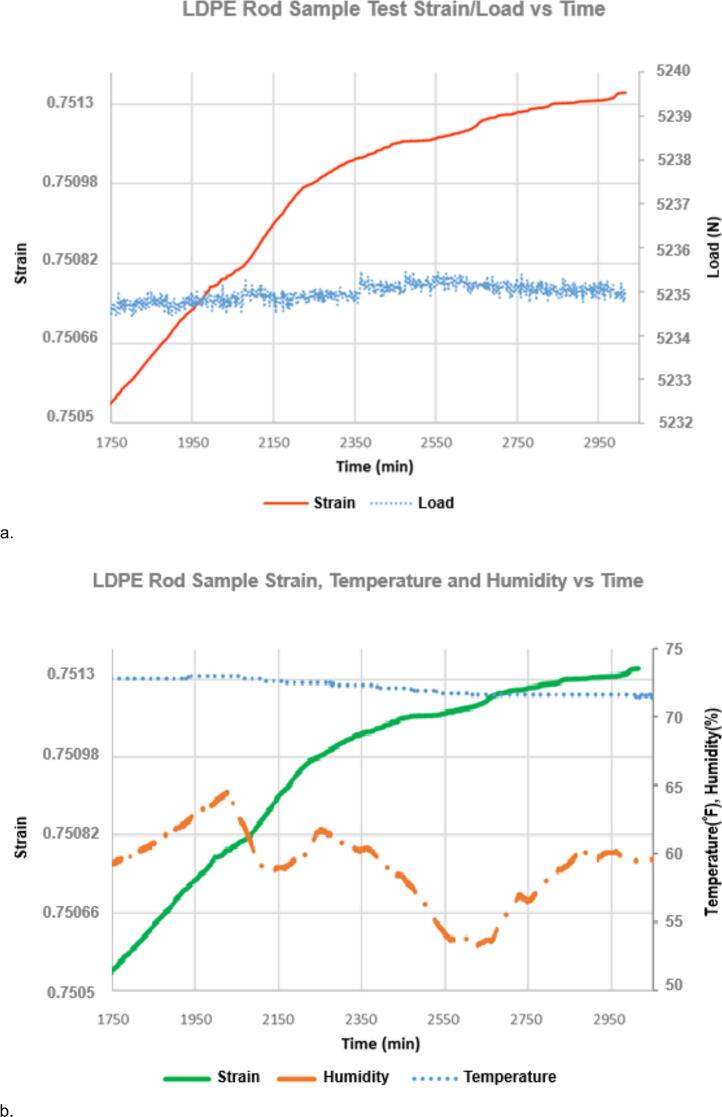
a. LDPE extruded rod sample creep vs load, b. corresponding temperature and humidity vs time. The shape of the travel vs. time data does not have a direct relationship to load, temperature, or humidity. Travel data points are collected once per minute so that the shape of the travel curve is due to measured data points and not due to a curve fit between sparse data points; the shape of the curve represents the actual behavior of the sample and is not due to random scatter of data points.

**Fig. 32:**
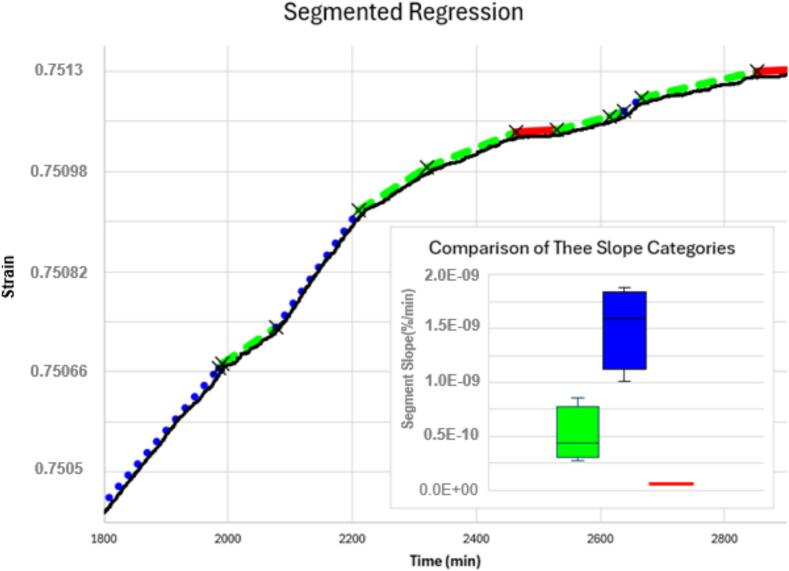
Extruded LDPE exhibiting segmented creep strain. The black line is experimental data measured with the Nano-Gauge. The segmented package [19] in R [20] was used to identify breakpoints and linear segments were fitted between the breakpoints. For easier viewing, the fit has been shifted upward slightly so that it parallels the experimental data instead of covering it. The x-marks indicate breakpoints between segments. The slopes of the segments can be divided into three distinct categories. The blue dotted segments represent the high strain rate category, the green dashed segment represents the medium creep rate category, and the red solid segments represent the slowest creep category. The inset boxplot shows how the slopes of the segments in this single run are distributed; there is a clear separation between the three classes of slope. There were not enough red segments to make a full box in the boxplot, but the separation into three categories based on slope is consistent with previous findings [1]. (For interpretation of the references to color in this figure legend, the reader is referred to the web version of this article.)

With the high precision and rapid sampling rate, creep curves collected with the nano gauge provide more information about creep behavior than conventional tests. The price of the nano gauge is more than 3 times the cost of the test rig, but it makes a valuable addition for advanced creep investigations. Fig. 31a and b show strain, load, temperature, and humidity data for a creep test on a low-density polyethylene (LDPE) extruded rod (McMaster-Carr, 8754K43). The 3/8-inch (0.9525 cm) diameter rod was cut to a height of 1.0592 cm (0.417 in.) using a sharp knife to ensure the ends were smooth and square. With lower resolution and lower sampling rates, the segmented shape of the curve could have been misinterpreted as “scatter” or random error, but as Fig. 31 shows, the behavior does not stem from variations in applied load, temperature, or humidity, but is due to the inherent behavior of the sample. Interestingly, we find that the slope of the LDPE sample can be fit using segmented linear regression, and the slopes found from the fit can be divided into three distinct classes which may represent distinct molecular-level creep mechanisms, see Fig. 32. A more detailed discussion of this behavior is provided in our recent publication [1].

## Ethics statements

Our work does not involve experiments on animals or humans.

## CRediT authorship contribution statement

**Seyyed Saeed Vaezzadeh:** Writing – original draft, Visualization, Validation, Methodology, Investigation. **Alireza Godsi Chafjiri:** Writing – review & editing, Investigation. **Victoria Nguyen:** Investigation, Funding acquisition. **Xochitl Ramirez:** Investigation. **Robert Kelley Bradley:** Writing – original draft, Visualization, Validation, Supervision, Methodology, Funding acquisition.

## Declaration of competing interest

The authors declare that they have no known competing financial interests or personal relationships that could have appeared to influence the work reported in this paper.
